# Early-life enrichment in American mink (*Neogale vison*): Enrichment of the perinatal environment improves maternal nest building and reduces stereotypic behaviour

**DOI:** 10.1017/awf.2024.71

**Published:** 2025-01-13

**Authors:** Gabrielle B Clark, María Díez-León, Rebecca K Meagher

**Affiliations:** 1 Dalhousie University Faculty of Agriculture, Department of Animal Science and Aquaculture, Truro, Nova Scotia, Canada; 2 Royal Veterinary College University of London, Department of Pathobiology and Population Sciences, London, UK

**Keywords:** abnormal behaviour, American mink, animal welfare, early-life environment, environmental enrichment, maternal care

## Abstract

Pens for farmed mink (*Neogale vison*) commonly include separate nesting areas to provide privacy and warmth in the perinatal period. However, standard bedding materials may not be sufficient to allow intrinsically motivated nest-building behaviours in dams. Further, these materials may not produce optimal nest structures for the rearing of kits. In the present study, we provided extra, relatively high-quality nest-building materials and a chewable sisal rope enrichment for mink dams in the perinatal period (a group enriched at whelping; EW). The effects of these enrichments on various measures of welfare and maternal behaviour were compared to those of mink dams in standard housing (SH) and mink dams whose kits were enriched later in development (EK). EW dams performed less stereotypic behaviour and built higher quality nests than dams of other housing conditions, although dams’ basal faecal cortisol metabolite levels (FCM) were not affected. The stress responsiveness of these dams’ offspring was later assessed by sampling FCM before and after a handling event, however, this event did not appear to induce a measurable stress response and thus no conclusions could be drawn regarding effects of perinatal enrichment on HPA-axis development. Overall, provision of higher quality nest-building materials and a chewable rope enrichment benefited dam stereotypic behaviour and nest building in the perinatal period. We present suggestions for future studies to further investigate whether perinatal enrichment can impact maternal care and offspring HPA-axis development in mink.

## Introduction

Farm animal welfare has been linked to aspects of housing such as feeding regimens (Rushen 1985; Lewis *et al.* 2022), access to social partners (or separation from others, if a solitary species; Nimon & Broom 1999), and adequate space or environmental resources for movement or travel according to what the species has evolved to do in the wild (Mason *et al.* 2001; Clubb & Mason 2007). Likewise, aspects of housing and husbandry for American mink (*Neogale vison*), a species farmed for their fur, have been adapted to better allow the expression of natural behaviours, in turn improving animal health and productivity. The Code of Practice requires that commercial mink in Canada must be provided access to a separate nesting area in the form of a nest-box to allow for privacy and warmth (National Farm Animal Care Council 2013); in the wild, mink will often make use of multiple underground dens scattered throughout their territory (Dunstone 1993). Although nest-boxes are generally provided year-round (for exceptions regarding temporary blocking or removal, see National Farm Animal Care Council 2013), bedding material for nest building and insulation must additionally be provided during whelping, lactation, furring, and winter months. This is especially important in the peri-whelping period because mink are altricial and born without the ability to thermoregulate (developed at 29 days of age), therefore hypothermia is one of the most common causes of kit mortality in the early postnatal period (Martino & Villar 1990). Provision of a nest-box is shown to reduce kit mortality and increase kit growth rate compared to litters raised without a nest-box (Møller 1990). Nest-box provision can also have stress-reducing effects for females prior to whelping and decrease their expression of stereotypic behaviour, i.e. invariant, repetitive, and apparently functionless patterns of motor behaviour (Hansen *et al.* 1994; Nimon & Broom 1999; Hansen & Jeppesen 2000). In turn, reduced physiological stress and stereotypic behaviour are known to correlate with improved kit-directed maternal care behaviour and nest construction (Malmkvist & Palme 2008; Schou *et al.* 2018).

However, there is a need for research regarding ways in which bedding materials and/or general nest environments for mink might be improved at whelping. Nest-boxes and bedding are standard provisions in most mink-farming countries prior to whelping (European Commission 2001; National Farm Animal Care Council 2013; State Forestry Administration of the People’s Republic of China 2016; Fur Commission USA 2019). European guidelines additionally recommend providing sufficient bedding material to build a closed nest in the box and to ensure the nest-box is protected from draughts (Møller *et al.* 2015); the bedding materials provided in Europe may include hay, straw, flax, shredded straw/paper, wood-shavings, wool, or other materials with insulating properties. In Canada, standard nest-building materials include wood-shavings, chopped straw, and hay, and current recommendations for farms specifically include packing nest-box corners with bedding and providing a nest-building material that facilitates a bowl shape to keep kits close together and improve nest temperature (National Farm Animal Care Council 2013). However, these guidelines do not specify which materials are favourable for insulation or construction of an enclosed nest, and standard bedding materials (particularly those provided in Canada) may be insufficient to form a nest capable of maintaining optimal temperatures. Kit mortality in farmed mink is considered similar to that of wild-living mink (estimated between 20 and 35% and 22 and 35%, respectively; European Commission 2001), but would ideally be improved since many of the survival challenges facing wild-living mink kits are absent in farmed settings, and reducing mortality would also be economically beneficial to farmers. It is also possible that lack of adequate nesting material may constrain behavioural opportunities for females, thus affecting their stress levels, stereotypic behaviour, and maternal care behaviour (Malmkvist & Palme 2008).

Limited access to straw for nest building has been shown to significantly reduce offspring weights, increase mortality of live-born kits, and increase maternal cortisol compared to groups with a pre-made plastic nest or plastic nest with straw (Malmkvist & Palme 2008). Motivation to perform maternal care behaviours may also be improved by increased nest-building opportunities, since dams with access to a plastic nest with straw were quicker to retrieve their kits in a kit retrieval test than those without these materials (Malmkvist & Palme 2008). Prolonged access to standard nest-building materials has also been shown to be beneficial: dams provided with nest-building materials in January showed greater reproductive success, measured by litter size and offspring survival, and reduced basal stress levels compared to dams who received materials in March (Schou *et al.* 2018). Moreover, Campbell *et al.* (2016) found that nests incorporated with wood-shavings were better constructed than nests made of chopped straw; however, they did not include measures of dam welfare or long-term effects on kits when each of these bedding materials were used. From this study, it was also concluded that chopped straw may be an ideal nest bottom substrate if additional materials such as uncut straw or wood-shavings are also provided to fortify the nest (Sønderup *et al.* 2009; Campbell *et al.* 2016). Thus, there is an opportunity for researchers to examine dams’ welfare and reproductive success when multiple standard and high-quality substrates are provided, including the quality of nest construction facilitated by these materials.

There has also been little investigation regarding the effects of prenatal or early postnatal stress, maternal care, and nest quality on development of stress responsiveness in farmed mink. It has been established in other mammalian species that prenatal stress and maternal stress during lactation can directly impact offspring stress responsiveness and long-term health, namely via the actions of maternal stress hormones on the developing fetus (for a review, see van Bodegom *et al.* 2017; Weinstock 2017) or by passage of maternal stress hormones through maternal milk (Stead *et al.* 2022). This is in addition to behavioural effects of maternal stress on quality of maternal care and resulting implications for offspring stress responsivity. It has been documented in many species (including rodents, pigs [*Sus scrofa*], non-human primates, humans, and dogs [*Canis familiaris*]; summarised in Lezama-García *et al.* 2019) that increased quality of nursing, licking, and grooming of offspring can improve stress resilience and mitigate anxiety- or depression-like phenotypes in the offspring as adults. For altricial dams (including mink) that also build nests leading up to parturition as a component of maternal care, nest-building behaviours can play a role in promoting long-term kit health since nest temperature is known to modulate offspring stress response development (Jans *et al.* 1985; Jans & Woodside 1990).

The present study was part of a larger experiment conducted on the same farm with the same cohort of mink, with objectives relating to enrichment of the physical environments of farmed mink at different life stages (see Clark *et al.* 2025 for the companion article to this study which has been published simultaneously). Here, we outline the objectives associated with the first part of this experiment: we aimed to determine if enriching the perinatal environment of farmed mink could positively modulate dam behaviour, welfare, and reproductive success, and deliver long-term benefits to kit stress responsiveness. It was hypothesised that dams provided with enriched nest-building materials and a physical, chewable enrichment in the peri-whelping period would demonstrate reduced basal faecal cortisol levels and reduced stereotypic behaviour compared to dams with standard nest-building materials and enrichment due to greater opportunities to express natural nest-building behaviour. Consequently, kits in enriched whelping environments were hypothesised to receive a higher quality of maternal care than kits reared in standard environments as an indirect result of decreased maternal stress and greater motivation to perform kit-directed maternal care behaviours. Improved nest construction in enriched whelping conditions was also predicted, and this factor, in combination with potential increases in quality of maternal care and reductions in prenatal stress, were predicted to benefit kit survival and stress responsiveness later in life (assessed via faecal cortisol responses following a stressor and post mortem spleen weights as an indicator of chronic stress effects; Díez-León *et al.* 2016).

## Materials and methods

### Subjects and housing

A total of 242 female mink were bred to account for potential unsuccessful copulations and/or poor litter health. Male and female mink selected at breeding for use in the study were balanced across Dark, Mahogany, Pastel, Demi, and Stardust colour types (strains). All dams were housed individually (adult American mink are solitary in the wild; Dunstone 1993) indoors at the Canadian Centre for Fur Animal Research (Nova Scotia, Canada) in 75 × 30 × 45 or 40 cm (length × width × height) wire-mesh pens with a wire shelf (25 × 30 × 25 cm; length × width × elevated), external wooden nest-boxes (25 × 30 × 20 or 18 cm; length × width × height), and a single plastic ring enrichment (3.8 × 10 cm; thickness × diameter) prior to assignment of their respective conditions. Mink were fed with a meat-based paste placed on the mesh roof of the pen; feedings took place once a day in the afternoon for non-reproductive adults and twice a day (morning and afternoon) for pregnant dams approaching parturition, lactating dams, and kits. All mink had *ad libitum* access to drinking water via automatic drinkers. The research was approved by the Dalhousie University Faculty of Agriculture Animal Care & Use Committee (#1033575) and the Clinical Research Ethics Review Board of the Royal Veterinary College (URN 2021 2034-3).

### Housing in the peri-whelping period

Dams who bred successfully (n = 242) were randomly assigned to one of three experimental groups: standard housing (SH; n = 59), enriched at whelping (EW; n = 119) or enriched once kits were mobile (EK; n = 64 [relevant to the companion article to this study; Clark *et al.* 2025]). Groups were balanced for colour type and parity and pens were evenly distributed throughout the barn to account for potential effects of variable lighting, temperatures, noise levels, etc. Dams assigned to the SH and EK housing conditions were given standard nest-building materials (chopped straw with wood-chip bedding) in the period leading up to whelping. Dams in EW were given standard nest-building materials in addition to a handful (~7.7 g) of crumpled tissue (Kaytee Clean & Cosy bedding, Chilton, WI, USA) to soften/insulate their nests, and a handful (~3.9 g) of excelsior curled aspen shavings (also known as wood wool) to fortify the structure of their nests. These materials were provided on the same date for all EW pens, thus access lasted a minimum of seven days and a maximum of 16 days prior to whelping due to variable whelping dates. A length of sisal rope (1.3 cm in diameter and 43 or 38 cm long, according to pen height) was also fixed to the ceiling in EW pens (provided a minimum of five days and a maximum of 14 days prior to whelping); this rope functioned as a hanging enrichment but could also be unwound and incorporated into nests by the dams, serving as additional nest-building material. These materials were replenished if the observer deemed that they had been soiled (i.e. contaminated with urine or faeces) or dropped through the pen bottom.

### Housing in the post-weaning period: Group housing

Kits were weaned at six weeks of age in accordance with standard farm protocols, at which point the dam was removed from the whelping pen and housed in a separate standard pen. Litters were excluded from further testing if fewer than four kits survived to this stage (number of litters after exclusions: n_SH_, n_EK_, and n_EW_ = 36, 33, and 47, respectively). Kits remained in the whelping pen and were housed in groups of four to six (n_SH_, n_EK_, and n_EW_ = 218, 183, and 207 kits, respectively); if there were more than eight kits in a litter, they were split across two pens (applied to ten SH pens, three EW pens, and seven EK pens). Enrichment provision for EK kits through this period is detailed in the companion article to this study (Clark *et al.* 2025) and in Clark *et al.* (2023) but, in brief, access to a hanging plastic chain (approximately 38–43 cm in length depending on cage height) and a standard plastic ring (previously described) was maintained in addition to the introduction of a second manipulable enrichment with benefits previously demonstrated in mink (a wiffle ball or golf ball). A schedule of enrichment exchange was implemented for EK kits such that mobile EEs were exchanged bi-weekly and hanging EEs were exchanged monthly to maintain object novelty. Access to a standard ring enrichment was maintained for SH and EW kits with no enrichment exchange.

### Housing in the post-weaning period: Pair-housing

At ten weeks of age (four weeks post-weaning), kits were moved to single- or pair-housing pens according to standard farm protocol. One male and one female from each litter were chosen for pair housing and remained in the whelping pen (dimensions of 75 × 30 × 45 or 40 cm [length × width × height]; n_SH_, n_EK_, and n_EW_ = 46, 37, and 42 pairs, respectively). A single female from each litter was moved to a drop-in cage, i.e. cages with a wooden nest-box at the back of the cage, connected at the ceiling of the cage so the mink are required to jump up into them (dimensions of 76 × 25 × 45 cm with a nest-box of 25 × 25 × 30 cm [both length × width × height]; n_SH_, n_EK_, and n_EW_ = 21, 23, and 27 females, respectively). Male-female pairs and single-housed females in EK continued to have access to rotating enrichments (a standard ring, a hanging EE in the form of a hanging sisal rope at this stage, and a second mobile EE in the form of a pig’s ear or hockey ball at this stage; for detailed methods, see Clark *et al.* 2023) until 15 weeks of age, at which point these were removed and only a standard enrichment remained. Male-female pairs and single-housed females in SH and EW maintained access to a standard ring enrichment throughout this period.

### Maternal behaviour observations

#### Dam behaviour scans pre-whelping

Instantaneous scan-sampling observations of all dams were conducted by an experienced observer with previous training in mink behaviour 3–5× per day for five consecutive days prior to whelping (within 6–22 days of dams’ whelp dates), and thus approximately 20 scans were collected per dam. All observations at this stage were conducted by the same observer. Observations began at least 30 min after the 0800h morning feeding (although mink typically eat in multiple short bouts throughout the day, most eat a first meal immediately after food delivery) and ended before 1200h in advance of afternoon feedings at approximately 1500h. The occurrence of stereotypic behaviour, lying awake, use of the rope enrichment (EW only), and resting were noted (for ethogram, see Table 1). The behaviour being exhibited by mink upon observation was recorded before moving to the next pen, with a 30-s habituation period if necessary (i.e. if mink appeared vigilant of the observer and/or if a behaviour could not be classified without prolonged observation, such as a stereotypic behaviour that must be repeated a certain number of times). One round of sampling took 60 (± 30) min, thus instantaneous scans for each dam were approximately 60 min apart.

**Figure 1.**
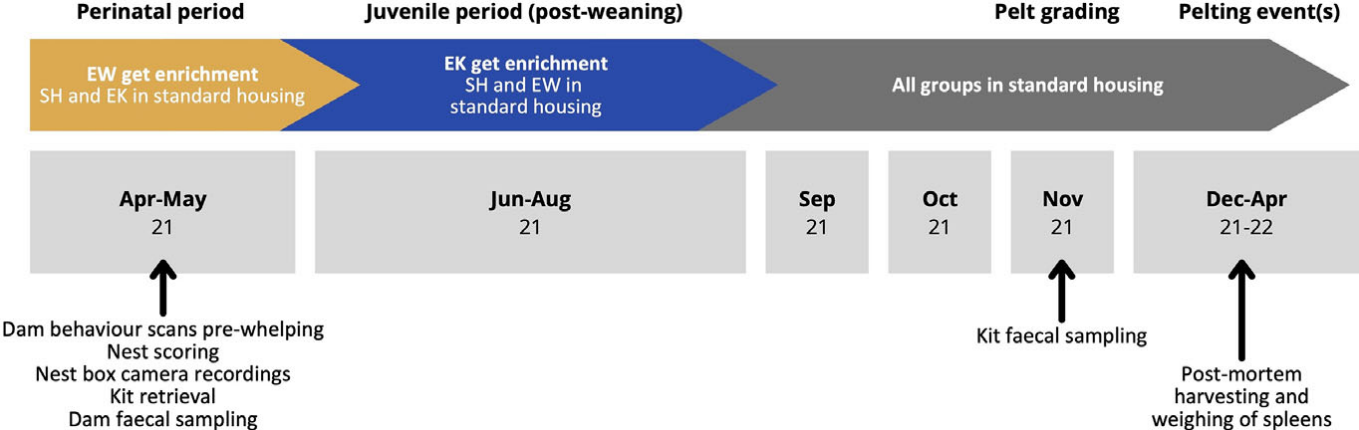
Timeline of standard mink-farming events taking place during the study (top), interventions for experimental groups (middle), and data collection for various tests (bottom). Months are indicated in grey boxes with the year (‘21’ denoting 2021 or ‘22’ denoting 2022).

**Table 1.** Ethogram for behaviour scans in farmed American mink dams (*Neogale vison*) included in study on maternal nest building and welfare

Behaviour	Description
Nest construction	Manipulating bedding materials with head/limbs or circling in nest forming a deeper bowl (circling must be repeated at least twice). Often performed in a scooping motion using the chin. Relevant in peri-whelping period only.
Stereotypic behaviour (SB)	Movements performed as three or more consecutive repetitions that are not necessary for eating, drinking or grooming. May be locomotory movement of the whole body, e.g. the mink pacing back and forth within the pen, or movement of isolated body regions, e.g. the mink moving the upper body or head from side to side (‘weaving’) or up and down the pen wall (‘twirling’; adapted from Díez-León *et al.* 2016; Polanco *et al.* 2017).
Borderline SB	Movement pattern resembling SB but interrupted before three repetitions, or switching occurs between elements of common stereotypies without repeating a sequence three times. Not analysed as SB but instead included in overall activity (e.g. Dallaire *et al.* 2011; Díez-León *et al.* 2016).
Resting	Inactivity with head down and eyes closed or hidden (adapted from Meagher *et al.* 2013).
Lying awake	Inactivity with eyes open (adapted from Meagher *et al.* 2013).
Inactivity	Lying motionless other than slight postural adjustments; category used if observer is unable to tell if resting vs lying awake (adapted from Meagher *et al.* 2013).
General activity	Engaged in activity not in any of the above categories; includes eating, drinking and grooming self.
Enrichment use	Head in contact, licking, chewing, or sniffing rope enrichment within 1 cm; excludes sleeping with the enrichment.

#### Nest scoring

Nests of all dams were scored visually for construction quality once per day, approximately five days per week throughout the whelping period (from 22 April to 5 May). Scores were collected by an experienced observer and an undergraduate research assistant trained on the nest-scoring protocol (note: neither observer could be blind to housing conditions, or the hypotheses being tested). Two training sessions (484 observations) were completed at the beginning of the nest-scoring period during which the research assistant scored nest structures alongside the experienced observer, and inter-rater reliability was assessed through percent agreement calculations (minimum agreement of 80%) and Cohen’s kappa (minimum score of 0.61–0.80). Any areas of discrepancy at this stage were discussed to reach agreement regarding future scoring. Nests were assigned a score on a scale from 1–7 (rating system described in Table 2). Nest scores on postnatal day (PND) -1 and PND 7 were identified for analysis with respect to whelping dates for each pen; pens were excluded from analyses if no scores were obtained within ±1 day of these dates. A rating for the incorporation of extra materials into the nest structure was also recorded for dams in EW (rating system described in Table 2).

**Table 2.** Scoring criteria for nest shape and nest material use in farmed American mink (*Neogale vison*) (modified from Malmkvist & Palme 2008; Meagher *et al.* 2012)

Nest shape score	Description
1	No nesting material.
2	Some nesting materials; flat, unstructured.
3	All nesting material; flat, unstructured.
4	Saucer-shaped indentation.
5	Round hole with a side higher than the dam when lying down (covering at least one side of box or 25% of circumference of nest).
6	Round hole with sides mostly higher than the dam when lying down.
7	Round hole with high sides and a ‘ceiling’ or overhang on at least one section; may have fur incorporated.

Nest material use score (EW)	Description
1	None of extra material taken into nest.
2	Some material used.
3	Most of material placed on top of nest-box and/or at least two types of enriched nesting material (paper, shredded aspen fibre, or rope) used in nest.
4	All or most (> 1/2) of two types of nesting material in addition to some (< 1/2) of a third type used and incorporated into the nest.

#### Nest-box camera recordings

Once dams had whelped, nest-box cameras (IP Bird Box Cameras with 32C video recorders, GolBong Technologies Co, Guangzhou, China) were installed in a random selection of pens balanced across conditions and evenly distributed throughout the barn (n_SH_, n_EW_, and n_EK_ = 9, 14, and 13, respectively). Cameras were mounted into the wood of the upper nest-box and angled downwards into the nest-box, with a plastic cover propped over the camera, to record activity while maintaining privacy for the nest. Activity was recorded for eight consecutive days post-whelping. Continuous video analysis was conducted *post hoc* to quantify time spent performing kit-directed maternal care behaviours, stereotypic behaviours (only scrabbling and wire gnawing were observed in video footage since camera views were restricted to the nest-box), nest construction, and time spent out of the nest-box (see Tables 1 and 3 for ethogram). Due to time constraints of research personnel and occasional equipment errors in the processing/recording of nest-box video, a sample of 6 h per day in 30-min periods across the odd hours of the day (0100–0130h, 0300–0330h, 0500–0530h, etc) were scored for a selection of two postnatal days within PND 1–4 and two days within PND 5–8; preliminary descriptive analyses were conducted on a sample of videos scored early in the development of methodology to inform the selection of observation days across these periods, and little difference was observed in the quantity of maternal care behaviour performed within PND 1–4 and 5–8. Further, Díez-León and Mason (2016) found that individual differences in maternal licking and grooming across mink dams were so stable in the first week post-partum that future studies could collect data over fewer days (e.g. even days only) to obtain a representative picture of maternal care. Selection of postnatal days for scoring was randomised using an online randomiser (www.random.org) when possible, i.e. when complete footage for all postnatal days was available. Scoring order by postnatal day and time of day was also randomised to account for observer fatigue and other observer effects. Scoring was conducted by an experienced observer and an undergraduate research assistant (both blinded to housing condition where possible, i.e. where EW bedding materials could not be seen). Observers were trained prior to the commencement of data collection using a subset of three videos (86 observations), at which point an inter-rater reliability score of greater than 80% agreement had been achieved for each behaviour code. Percent agreement was calculated as ([# agreements/# total observations] × 100); criteria for an ‘agreement’ included use of the same behavioural code, the same behaviour start time within 5 s, and the same behaviour end time within 5 s according to the video time stamp. Footage for several pens could not be analysed due to missing or non-continuous footage on the required days of observation (i.e. equipment and/or internet connectivity issues), resulting in a final sample of five SH pens, nine EW pens, and nine EK pens used in analyses.

**Table 3.** Ethogram for maternal care in nest-box camera recordings of farmed American mink (*Neogale vison*) included in study on maternal nest building and welfare

Behaviour	Description
Kit retrieval	Bringing a kit from outside the nest area back into the nest, whether by using head/mouth or limbs.
Nursing	Kit’s head/mouth in contact with teats. If view of kit’s head is obstructed, can be evident from suckling motion in belly and pawing at the teat with forepaws. Bout marked as finished if behaviour is obscured for more than 5 s.
Licking and grooming	Anogenital region - Licking or gently biting kit’s anogenital area. Bout marked as finished if behaviour is halted for more than 1 s.
	Body or head region - Licking or gently biting kit’s body or head. Bout marked as finished if behaviour is halted for more than 1 s. If target area of grooming is obscured but grooming motions are clear, list as body grooming (rather than anogenital or head).
Out of nest-box	Dam leaves the nest-box, behaviour not visible. Re-entry occurs when all four paws are across the threshold of the nest-box.

#### Kit retrieval

Kit retrieval tests were conducted with a random subset of dams balanced across housing condition, colour type, and parity (n_SH_, n_EK_, and n_EW_ = 34, 32, and 45, respectively) at 7 (± 2) days post-whelping by an experienced observer and an undergraduate research assistant. This test involved removing one kit from the nest and placing them in the main pen area facing the nest-box entrance (see Malmkvist & Houbak 2000; Meagher *et al.* 2012). Male and female kits were selected for removal on an alternating basis whenever possible to control for sex effects on retrieval latency. The latency of the dam to touch (i.e. come within 1 cm of the kit) and retrieve the kit (i.e. return them to the nest) was recorded. Dams were given a maximum of 180 s to retrieve kits, and if not retrieved by this time, they were recorded as not retrieving the kit and excluded from analysis.

### Kit mortality and growth data collection

Kits were counted and weighed by farm staff at PND 1 (‘first weight’; used to account for kits not born live), three weeks of age, and at weaning. Using these data, kit mortality across litters of different housing conditions was assessed from first weight to three weeks and from first weight to weaning. Litters were excluded from analysis if foster kits were added to the litter at any point (fostering was avoided in trial litters whenever possible, though five EW litters and three EK litters were excluded for this reason). Average kit weights at three weeks and at weaning were also assessed using these data. Likewise, causes for exclusion included kits being fostered into the litter or loss of all kits in the litter by the time of weight recordings.

### Physiological measures

#### Dam faecal sampling and cortisol extraction

To compare basal cortisol levels of dams across groups, faecal samples were collected from a random subset of dams balanced across housing conditions and locations in the barn (n_SH_, n_EK_, and n_EW_ = 44, 36, and 57 dams, respectively). Sample dates were adjusted according to each dam’s whelp date to ensure sampling during similar biological states (approximately 20 days post-whelping); mesh screens with wooden frames to collect faeces were placed below pens beginning at 1000h and retrieved within 2 h of that time the next day. Samples were frozen until later processing to assess levels of faecal cortisol metabolites (FCM) with a mink-validated 11ß-hydroxyaetiocholanolone enzyme immunoassay (Malmkvist *et al.* 2011). The loss of several samples during transport for processing resulted in a final sample of 29 SH dams, 27 EK dams, and 41 EW dams for analysis.

#### Kit faecal sampling and cortisol extraction

To assess differences in stress physiology of kits across groups, faecal samples were collected from a subset of male-female pairs (n_SH_, n_EK_, and n_EW_ = 29, 29, and 32 pairs, respectively) preceding and following pelt grading. Pelt grading was used as a stress event since it is practiced annually on commercial farms, and by substituting this event for experimental restraint stress in carrying cages (typically used to induce stress in mink; Malmkvist *et al.* 2011), we aimed to avoid subjecting mink to additional stress. Mesh screens with wooden frames were placed below pens for pre-test faecal sample collection two days prior to pelt grading at approximately 1400h and collected the following day between 1000–1300h. On the testing day, pelt grading took place from approximately 0800–1000h; screens were put in place for post-test faecal sample collection 4 h following pelt grading (a time lag shown to reflect cortisol excretion in mink faeces; Malmkvist *et al.* 2011) at approximately 1400h and collected the next day between 0930–1230h. Faecal samples were frozen for later FCM extraction using a mink-validated 11ß-hydroxyaetiocholanolone enzyme immunoassay (Malmkvist *et al.* 2011).

#### Post mortem harvesting and weighing of spleens

The spleens of pair-housed males were harvested upon pelting (n_SH_, n_EK_, and n_EW_ = 17, 18, and 16 males in January pelting; an additional sample of n_SH_, n_EK_, and n_EW_ = 8, 8, and 10 males were added in April pelting). Spleens were trimmed of fat and weighed; weights were then compared across groups. Bodyweights and lengths of mink were also recorded to use as controls in analyses if needed.

### Statistical analysis

All statistical analyses were conducted with jamovi statistical software (the jamovi project 2023; v. 2.3.18.0 for Mac). Figures were generated using Prism (GraphPad Software 2023; v 10.02 for Mac). Significance level was set at *P* < 0.05. Results were defined as tendencies when 0.05 < *P* < 0.10. Assumptions of normality and homogeneity of variances for parametric analyses were assessed using Shapiro-Wilk and Levene’s tests, respectively. Transformations were performed as necessary (either square-root transformations or log_10_ transformations, as appropriate) with mean and 95% confidence interval (CI) subsequently back-transformed for presentation. Where parametric analyses were not appropriate, non-parametric alternatives were used.

#### Analysis of housing effects on dam welfare indicators

For tests occurring prior to postnatal week three, SH and EK housing conditions were pooled for analysis (henceforth referred to as SH&EK) as they were in equivalent housing at this time. Behavioural scan data pertaining to dam stereotypic behaviour, resting, lying awake, and interaction with the rope EE were analysed as counts of behaviours across conditions using Poisson or Quasi-Poisson regressions (as appropriate based on assumptions for the model); dam colour type and parity were not included in this model as these factors were balanced across pooled conditions. Rope use in EW was assessed qualitatively as no other groups had this enrichment. Basal FCM in ng g^–1^ of SH&EK dams and EW dams were compared using a Student’s *t*-test, though were log_10_ transformed for analysis due to non-conformity with the normal distribution and subsequently back-transformed for presentation.

#### Analysis of housing effects on maternal care and nest building

Maternal care behaviours were formatted as a percentage of time for analysis (total cumulative time spent performing behaviour/total time observed × 100). Average bout durations of each behaviour were also analysed (total cumulative time spent performing behaviour/total number of occurrences); these bout durations were presented in minutes (min) or seconds (s) as appropriate. Average percentages of time and average bout durations were then compared across EW and SH&EK dams using Student’s *t*-tests when assumptions of normality and homogeneity of variances were met, or non-parametric Mann-Whitney *U* tests when data were not normally distributed or when transformations were not successful. Dam parity and litter size were not included as factors in these models because parity was balanced across the pooled conditions, and litter sizes did not significantly differ between pooled conditions; moreover, the non-parametric models used did not allow for the inclusion of additional blocking factors. Latency of SH&EK dams to touch and retrieve kits (s) was compared to that of EW dams using Student’s *t*-tests. A two-way ANOVA with housing condition and dam colour type as factors was also conducted to determine effects of dam colour type on retrieval latency due to previous evidence of colour effects on this measure (Clausen *et al.* 2008); it was determined in preliminary analyses that kit touch and retrieval latencies did not differ by kit sex (overall average of 23.6 [± 30.1] and 42.7 [± 37.0] s for females and 24.7 [± 29.1] and 43.4 [± 34.5] s for males, respectively), so it was not controlled for in analyses. Nest scores for SH&EK dams and EW dams were compared using non-parametric Mann-Whitney *U* tests as subjects were assigned scores on an ordinal ranking system. Nest scores on PND -1 and PND 7 were assessed in separate models. Frequencies of material use scores and combinations of materials used in EW dams were assessed using descriptive statistics.

#### Analysis of housing effects on kit mortality and growth

Percent mortality at three weeks and at weaning were compared across housing conditions using a non-parametric Mann-Whitney *U* test and Kruskal-Wallis one-way ANOVA, respectively. Litter size was not included as a factor in analyses because percent mortality was used as a standardisation method, and litter sizes in our study sample were constrained to relatively average litters (a maximum of 12 live kits with a mean of 5.29 kits in SH&EK and 4.43 kits in EW; median of five kits in both groups). Moreover, the non-parametric models used did not allow for the inclusion of additional blocking factors for this measure. Average kit weights at three weeks and at weaning were compared using Student’s *t*-test and one-way ANOVA (Welch’s) with housing condition as a factor.

#### Analysis of housing effects on kit stress responsiveness and chronic stress effects

Kit pre- and post-test FCM in ng g^–1^ for each housing condition were analysed using paired Student’s *t*-tests. Correlation of male spleen weights with bodyweight and body length were assessed using descriptive scatterplots. When it was determined that these did not correlate positively for all males and body weight/length were not needed as covariates in the analysis, spleen weights were analysed using one-way ANOVA (Welch’s) with housing condition as a factor.

## Results

### Housing effects on dam welfare indicators

**Figure 2.**
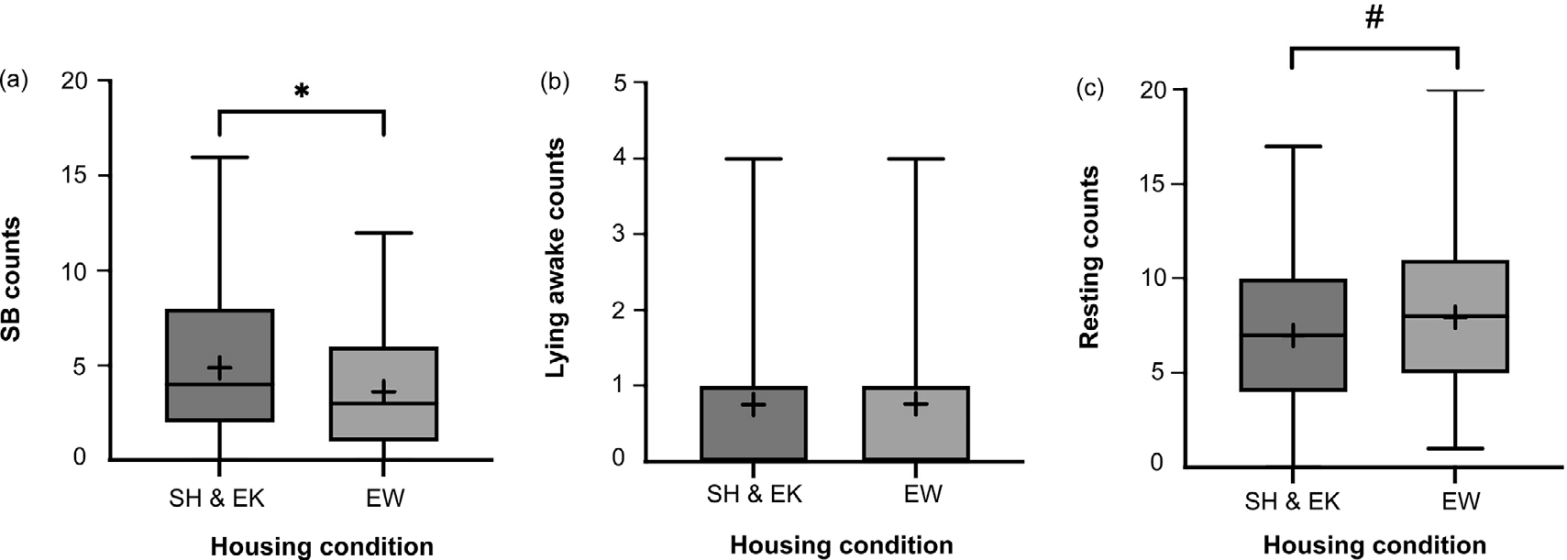
Box and whisker plots of counts (observations) where the following behaviours occurred in farmed American mink (*Neogale vison*) dams of the standard housed (SH) and enriched kits’ (EK) conditions pooled for comparison to dams in the enriched at whelping (EW) condition: (a) stereotypic behaviour (SB), (b) lying awake, and (c) resting. Black + signs show the means. N = 123 and 119 sample points, respectively, for each figure. Bars with * indicate a significant difference (*P* < 0.05) and those with # indicate a non-significant tendency (0.05< *P* < 0.10).

EW dams performed significantly fewer stereotypic behaviours (SBs) than SH&EK dams (χ^2^_1_ = 7.63; *P* = 0.006; Figure 2). A tendency towards increased resting was observed in EW dams compared to SH&EK dams (χ^2^_1_ = 3.51; *P* = 0.061; Figure 2), though this difference was not significant. Lying awake was not affected by housing (χ^2^_1_ = 0.01; *P* = 0.945; Figure 2). Dams in the EW condition used the hanging rope enrichment in 0.681 (± 0.650) observations on average, though this behaviour was quite variable (minimum and maximum observation counts of 0 and 3, respectively). Dams in SH&EK and EW housing had similar basal FCM (back-transformed mean: 25.70 ng g^–1^, 95% CI [21.88, 29.51] and 28.18 ng g^–1^, 95% CI [23.44, 34.67], respectively), thus there was no effect of housing on this measure (*t*_95.0_ = 0.904; *P* = 0.368).

### Housing effects on maternal care and nest building

**Figure 3.**
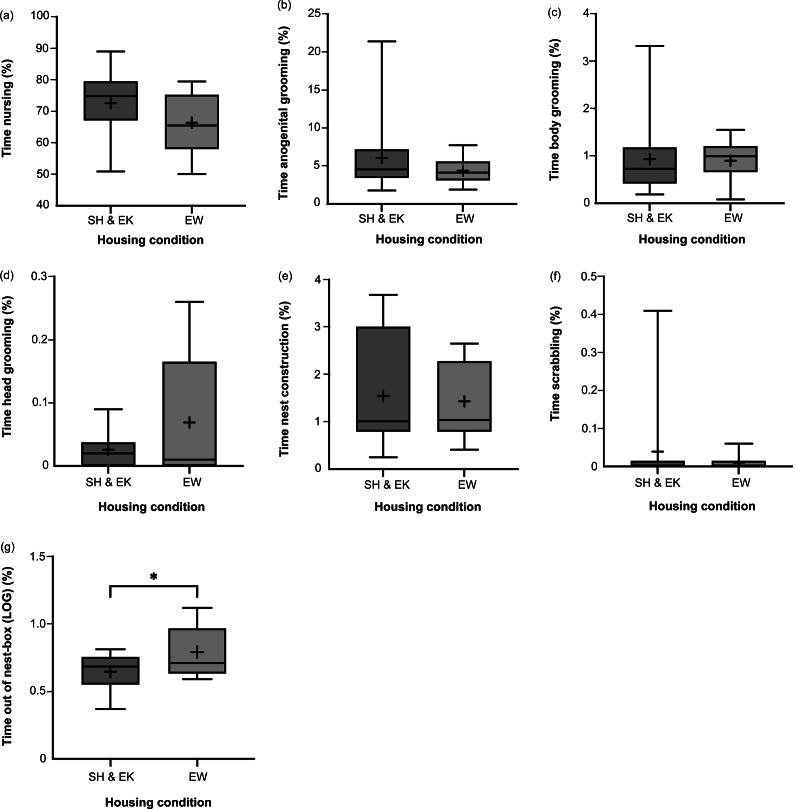
Box and whisker plots of percentage of time farmed American mink (*Neogale vison*) dams in the standard housed and enriched kits’ (SH&EK) conditions and enriched at whelping (EW) condition were observed performing the following behaviours: (a) nursing kits, (b) licking and grooming kits’ anogenital region, (c) licking and grooming kits’ body region, (d) licking and grooming kits’ head region, (e) nest construction, (f) scrabbling, and (g) out of the nest-box. N = 14 and 9 sample points, respectively, for all figures. Black + signs show the means. Bars with * indicate a significant difference (*P* < 0.05).

**Figure 4.**
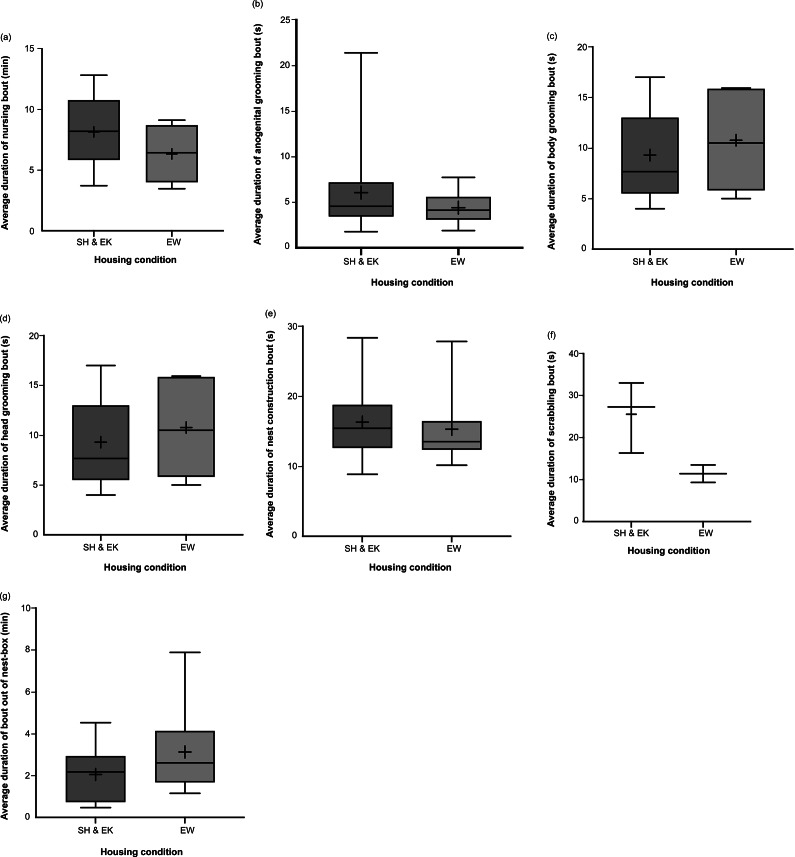
Box and whisker plots of average duration of bouts of the following behaviours across farmed American mink (*Neogale vison*) dams in the standard housed and enriched kits’ (SH&EK) conditions and enriched at whelping (EW) condition: (a) nursing kits, (b) licking and grooming kits’ anogenital region, (c) licking and grooming kits’ body region, (d) licking and grooming kits’ head region, (e) nest construction, (f) scrabbling, and (g) out of the nest-box. N = 14 and 9 sample points, respectively, for (a)–(c), (e) and (g); n = 11 and 5 sample points for (d), and n = 3 and 2 sample-points, respectively, for (f). Black + signs show the means.

Time spent nursing, anogenital grooming, body grooming, and head grooming were not affected by housing condition, nor were average bout durations of these behaviours (*P*-values ranged from *P* = 0.117 for *t*_21.0_ = –1.634 to *P* = 1.000 for *U* = 62.5; Figures 3 and 4). Time spent performing nest-construction behaviours and average nest-construction bout durations were similar across groups (*U* = 63.0; *P* = 1.000 and *t*_21.0_ = –0.448; *P* = 0.659, respectively). Time spent scrabbling and average scrabbling bout duration also did not differ by housing (*U* = 0.943; *P* = 0.896 and *t*_3.00_ = –2.173; *P* = 0.118, respectively); measures of wire-gnawing behaviour could not be analysed due to a lack of this behaviour in SH dams and EW dams. However, EW dams spent greater time out of the nest-box compared to SH and EK dams (back-transformed percentage of time: 6.18%, 95% CI [4.42, 8.65] and 4.43%, 95% CI [3.71, 5.28], respectively; *t*_21.0_ = 2.167; *P* = 0.042; Figure 3). Average out-of-nest-box bout duration was not affected (*t*_21.0_ = 1.561; *P* = 0.133; Figure 4).

Latency to touch and retrieve kits did not differ by housing condition (*t*_95.0_ = 0.265; *P* = 0.792 and *t*_92.0_ = –0.509; *P* = 0.612, respectively); SH&EK dams had latencies of 13.49 s (95% CI [10.00, 17.78]) to touch and 32.36 s (95% CI [25.70, 40.74]) to retrieve their kits (back-transformed), while EW dams had latencies of 14.13 s (95% CI [10.00, 19.95]) to touch and 25.70 s (95% CI [22.39, 38.90]) to retrieve their kits (back-transformed). Moreover, neither measure differed by dam colour type (*F*_3,89_ = 0.575; *P* = 0.633 and *F*_3,86_ = 0.841; *P* = 0.475), and there was no interaction effect between dam colour type and housing (*F*_3,89_ = 0.470; *P* = 0.704 and *F*_3,86_ = 0.329; *P* = 0.804).

**Figure 5.**
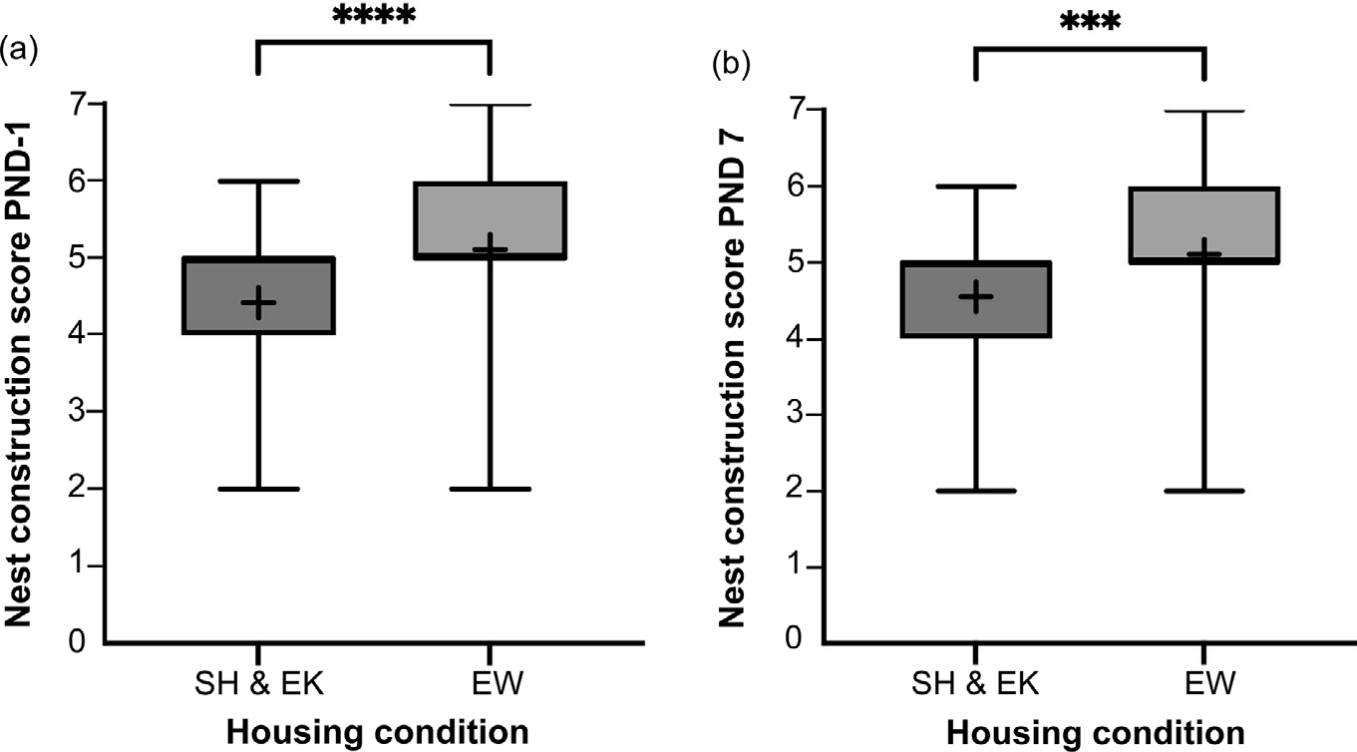
Box and whisker plots of average nest construction scores across farmed American mink (*Neogale vison*) dams in the standard housed and enriched kits’ (SH&EK) conditions and enriched at whelping (EW) condition on (a) PND -1 (n = 123 and 118 sample points, respectively) and (b) PND 7 (n = 121 and 118 sample points, respectively). Black + signs show the means. Bars with * indicate a significant difference (**** indicating *P* < 0.0001 and *** indicating *P* < 0.001).

Nests of EW dams were rated significantly higher than those of SH&EK dams on PND -1 (*U* = 5,126; *P* < 0.0001) and on PND 7 (*U* = 5,185; *P* < 0.001; Figure 5). A material use score of 3 (n = 40 dams) was the most common in EW by PND 7, with a score of 5 being the second most common (meaning all materials were incorporated into the nest; n = 37 dams). The entirety of the crumpled paper, curled aspen shavings, and rope were used in combination most often (n = 43 dams), followed by crumpled paper and aspen shavings (n = 34 dams) and crumpled paper and rope (n = 16 dams). Only one dam used a combination of curled aspen shavings and rope. In dams that made use of only one EW material, the crumpled paper was used most often (n = 12 dams).

### Housing effects on kit mortality and growth

**Figure 6.**
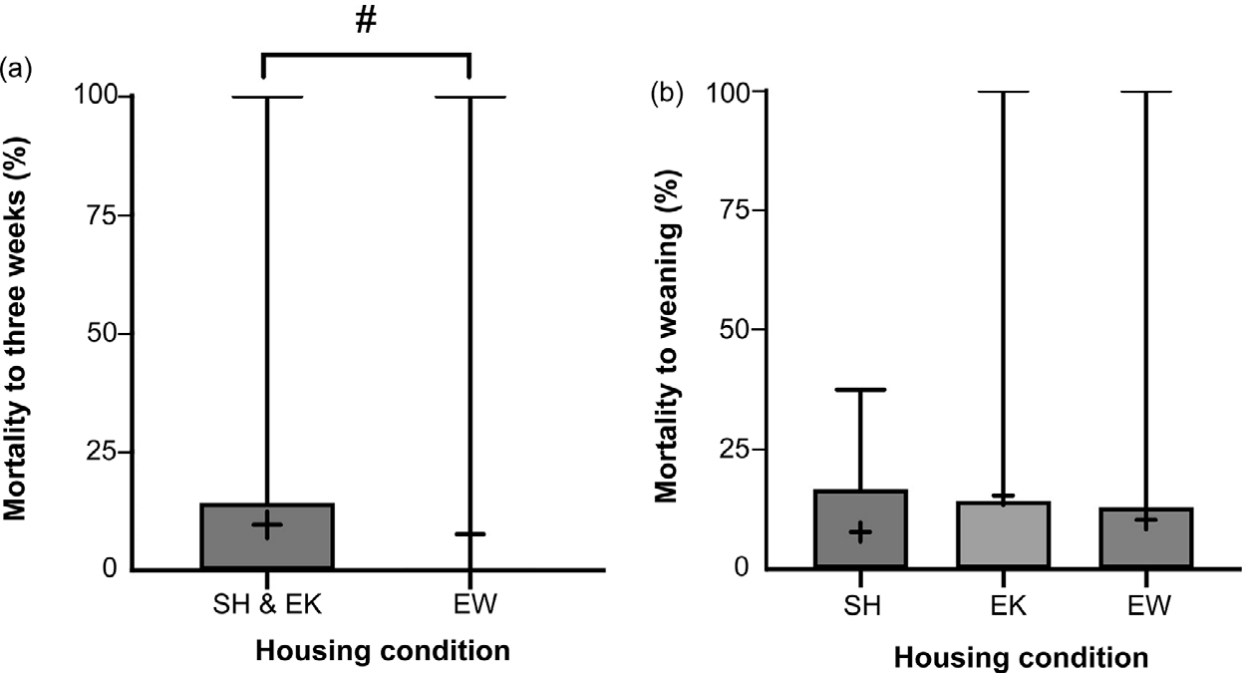
Box and whisker plots of percent mortality of farmed American mink (*Neogale vison*) kits from (a) first weight to three weeks between standard housed and enriched kits (SH&EK) and enriched at whelping (EW) (n = 95 and 82 sample points, respectively), and (b) first weight to weaning across litters of different conditions (n = 48, 47, and 82 sample points, respectively). Black + signs show the means. Bars with # indicate a non-significant tendency (0.05 < *P* < 0.10).

In the period from first weight to three weeks, EW litters tended to have lower percent mortality than SH&EK litters (*U* = 3,421; *P* = 0.075; Figure 6), though this difference was not significant and therefore no conclusions can be drawn about the effects of EW nest materials on kit mortality. There was also no difference in percent mortality from first weight to weaning (χ*^2^_2_* = 1.67; *P* = 0.435). Average kit weights at three weeks were 119 (± 2.57) and 118 (± 2.75) g in SH&EK litters and EW litters, respectively, thus there was no effect of housing condition (*t*_171_ = 0.197; *P* = 0.844). Likewise, average kit weight at weaning did not differ by housing condition (*F*_2,98.9_ = 0.490; *P* = 0.614); kits in SH, EK, and EW had average weights of 395 (± 10.95), 401 (± 13.05) and 386 (± 9.31) g, respectively.

## Housing effects on kit stress responsiveness and chronic stress effects

**Figure 7.**
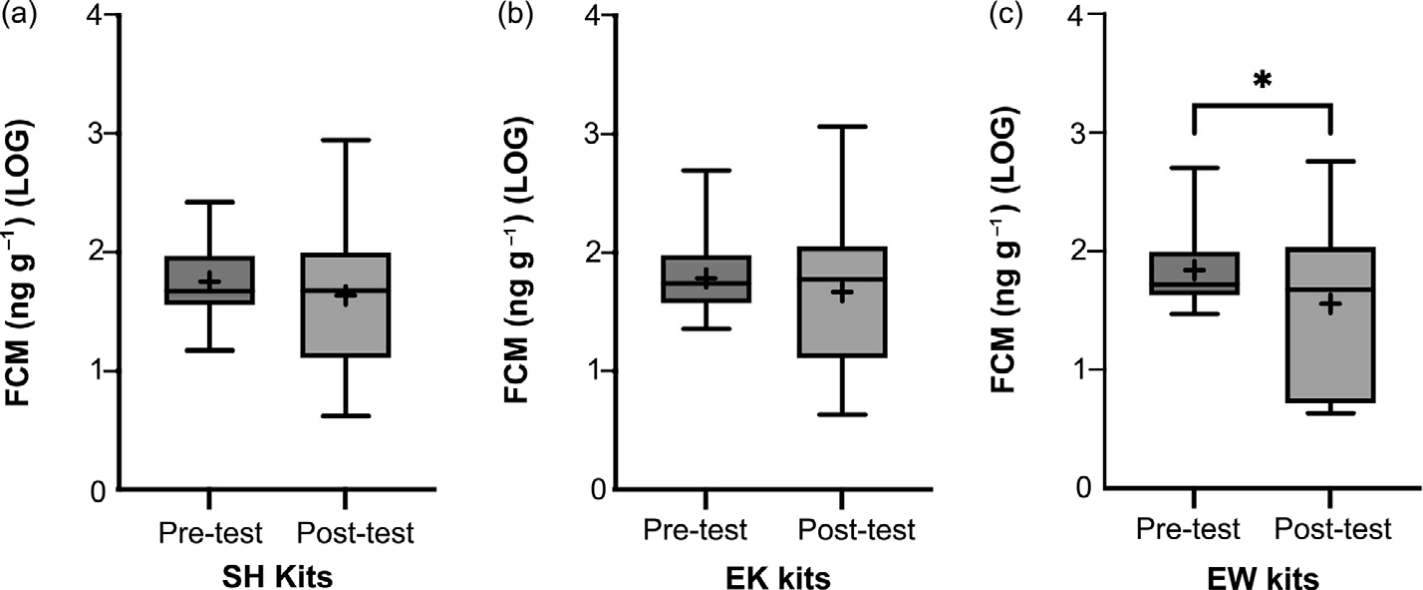
Box and whisker plots of log-transformed pre- and post-test faecal cortisol metabolite (FCM) concentrations (ng g^–1^) in farmed American mink (*Neogale vison*) kits of the (a) standard housed (SH) condition (n = 29 pairs), (b) enriched kits’ (EK) condition (n = 29 pairs), and (c) enriched at whelping (EW) condition (n = 32 pairs). Black + signs show the means. Bars with * indicate a significant difference (*P* < 0.05).

There was no difference in pre-test (i.e. basal) FCM levels across kits of different housing conditions (χ^2^_2_ *=* 1.813; *P* = 0.404). Similarly, there was no difference in pre- vs post-test FCM in SH kits (*t*_27.0_ = 0.714; *P* = 0.481) or in EK kits (*t*_28.0_ = 0.935; *P* = 0.358; Figure 7). However, EW kits had significantly decreased FCM in the post-test period (back-transformed mean: 33.11 ng g^–1^, 95% CI [19.50, 56.23]) compared to the pre-test period (70.79 ng g^–1^, 95% CI [53.70, 95.50]; *t*_31.0_ = 2.655; *P* = 0.012; Figure 7). Spleen weights did not differ between SH males (back-transformed mean: 7.14 g, 95% CI [5.97, 8.53]), EK males (7.04 g, 95% CI [5.85, 8.49], or EW males (7.40 g, 95% CI [6.32, 8.65]; *F*_2,49.0_ = 0.097; *P* = 0.908).

## Discussion

Despite a lack of evidence supporting the predicted impacts of EW housing on physiological measures of welfare (e.g. reduced basal faecal cortisol in EW dams, reduced post-stress faecal cortisol responses in EW kits, or reduced spleen weights in EW males as adults), significant effects of EW housing on dams’ stereotypic behaviour and nest quality were demonstrated. There was also some indication of nest material effects on dam resting behaviour and kit mortality that warrant further investigation. Possible explanations for these variable results are discussed below.

In support of our hypotheses for the EW intervention, there was a reduction in stereotypic behaviour observed in EW dams in the period leading up to whelping. Dams’ SB may have been reduced through multiple avenues; the time available for SB performance may have been directly limited due to time spent interacting with the rope enrichment and/or extra nest-building materials, or the internal drive to perform SB (if frustration-induced; Mason *et al.* 2007) may have been reduced by greater opportunity to perform motivated nest-building behaviours. Other hypotheses for underlying causes of SB, such as boredom (Mason & Latham 2004; Wemelsfelder 2005), may also have been addressed by the greater variety of stimuli to interact with in EW housing. However, lying awake, a behaviour that has been hypothesised to be associated with boredom (in mink; Meagher & Mason 2012, but see Polanco *et al.* 2021; in dogs; Harvey *et al.* 2019) was not affected. From rodent research, lying awake is also proposed to be associated with depression-like states as behavioural passivity in response to adverse situations (in mice [*Mus musculus*]; MacLellan *et al.* 2022), and EW housing had no effect on basal cortisol in dams in the peri-whelping period; thus, it is unlikely that depression-like states or other conditions associated with HPA-axis activation were mitigated by EW housing. Resting, meanwhile, is associated with positive welfare (distinct from lying awake in that the subject’s eyes are closed; for a review, see Fureix & Meagher 2015). Although our result for this measure was non-significant based on the model used to account for over-dispersion of the data (Quasi-Poisson regression), with greater statistical power this tendency may be revealed to be significant. As no other studies have looked at resting in mink when provided with extra nesting materials, this effect would be worth examining further.

Nest-construction scores of EW dams were also improved compared to dams of other conditions (similar to effects shown in Díez-León & Mason 2016). This difference reflects the achievement of walled nests (higher on all sides than the dam when lying down) with a partial overhang in EW pens, whereas SH and EK dams were not able to achieve these overhangs. EW dams most often made use of all the materials provided (including the sisal rope), incorporating the entire amount into their nests. It is therefore possible that the improved materials provided to EW dams facilitated this improvement in nest construction, while standard chopped straw and wood-shavings alone may be more limiting to nest shape. These results also demonstrate that dams will readily make use of the additional bedding materials provided in this study. We did not collect direct measures of nest temperature, but it is reasonable to believe that incorporation of these materials and improved nest construction would have benefited nest temperatures based on previous studies demonstrating the co-occurrence of high walled, roofed nests and higher nest temperatures (Malmkvist & Palme 2008; Schou *et al.* 2018).

It was also predicted that increased behavioural opportunities for EW dams would positively impact kit-directed maternal care behaviour, including behaviours like nursing, grooming, and retrieving kits. However, the only measure of maternal care behaviour impacted by EW housing was time spent out of the nest-box by dams (i.e. time where kit-directed behaviours were lacking). This result may reflect increased time spent interacting with the rope in the pen area; EW dams were occasionally recorded re-entering the nest-box with unwound rope fibres or tugging on their rope enrichments from the nest-box entrance, though it is uncertain whether this was always the case since the perspective of video footage was limited to the nest-box. This could be viewed as an interruption to maternal care caused by maternal enrichment, however, other maternal care behaviours were not affected as they were in another study that claimed a negative impact of EE on maternal care (Li *et al.* 2016). It should also be noted that spending time away from kits has been shown to be beneficial for dams, particularly as kits age (e.g. reductions in dam SB; Hansen 1990; Jeppesen 2004; Buob *et al*. 2013; Dawson *et al.* 2013; increases in time spent resting; Dawson *et al.* 2013; decreases in symptoms of mastitis; Buob *et al.* 2013), and therefore increases in time spent away from kits may indirectly improve the maternal care behaviours dams perform by improving their welfare.

It could in fact be inferred from EW dams’ increased time out of the nest-box that they were more efficient in their maternal care, spending similar amounts of time performing kit-directed behaviours while in the nest-box relative to SH and EK dams. This is further supported by the distinction made between ‘quantity’ and ‘quality’ of maternal care in other mammalian maternal care studies (defined as time physically spent with offspring and frequency of affiliative interactions with offspring, respectively; Aspillaga-Cid *et al.* 2021). While quantity, or time spent with offspring, was reduced in EW, time spent performing active maternal care behaviours was not significantly impacted. This replicates the results of Díez-León and Mason (2016), where additional enrichment for dams did not impact levels of kit-directed licking and grooming. Thus, SH and EK dams may have spent greater proportions of their time in the nest-box performing non-maternal behaviours compared to EW dams. It could also be interpreted that EW dams were exhibiting increased effort to reinforce/maintain the nest structure based on anecdotal evidence of them re-entering the nest with rope from the pen area, thus also spending their out-of-nest-box time performing maternal behaviour to some extent. Although measures of nursing sickness and/or mastitis were not collected in this study, it is also possible that greater time spent away from kits could have benefitted dams through the prevention of excessive suckling of kits (Buob *et al.* 2013; Dawson *et al.* 2013). Moreover, the tendency towards decreased kit mortality observed from first weight to three weeks in EW litters suggests that the EW intervention may have some benefit for reproductive success, despite no significant changes in observed maternal care; or at least, EW nesting materials did not increase kit losses. This effect on kit mortality may be revealed as significant if further, farm-wide studies using larger sample sizes are conducted with similar housing interventions.

It should also be noted that our sample size for these measures of maternal care was reduced due to technical difficulties encountered with the video equipment, which limited statistical power to detect differences. Recording of time spent nursing may also have been confounded by the quality and perspective of nest-box camera footage, as kits were often not visible due to the dams’ nursing postures (active bouts of nursing were only recorded when at least one kit could be seen attached to the nipple, or when kits were presumed to be suckling based on body position if the head was out of view). Similarly, bouts of nursing behaviour may have appeared shorter in dams who adjusted their posture more frequently, as kits would briefly appear in view before being concealed again.

Despite this limitation, the data collected are interesting in that it seems mink dams nurse almost continuously throughout the day (occupying approximately 70% of their observed time budget, and this may be an under-estimation). Patterns and circadian rhythm effects on maternal care are not well understood in mink, unlike other species whose maternal care is thoroughly documented (e.g. rabbits [*Oryctolagus cuniculus*] are known to nurse offspring roughly twice per day in bouts of less than 10 min each, while mice perform 25–35 nursing bouts of roughly 20–30 min per day, and distinct sequences of maternal care have been identified for each species; Jilge & Hudson 2001; Champagne *et al.* 2007; González-Mariscal *et al.* 2016). Such documentation of maternal care behaviours is useful in identifying whether dams are providing high- or low-quality maternal care, based on what is standard for the species. For example, assessment of ‘fragmented’ patterns of maternal care, which are known to have negative consequences for offspring HPA-axis development in rodents (Ivy *et al.* 2008; Couto-Pereira *et al.* 2016; Molet *et al.* 2016), is only possible when species-specific sequences of maternal care have been determined. It could also be investigated in future studies whether less frequent nursing or nursing in shorter bouts is indicative of higher quality milk production (i.e. milk of higher caloric content). Previous studies in mink have successfully quantified protein and fat content of maternal milk across post-partum weeks (Fink *et al.* 2001; Tauson *et al.* 2004), and similar methods could be applied to determine if enrichment of the dam increases milk quality, thus increasing kit weights despite reduced frequencies of nursing bouts or nursing bout durations, as has been found in rodents (DeRosa *et al.* 2022).

In video recordings with limited visibility, such as those used in the present study, more information regarding quality of nursing could potentially be derived by categorising the nursing postures of dams. There has been extensive research on nursing postures which are beneficial for offspring development in rodents, such as arched-back nursing (Myers *et al.* 1989) which is also known to be highly correlated with licking and grooming of pups (Meaney 2001), and its proposed analogue in dogs, vertical nursing (Bray *et al.* 2017). Meanwhile, maintaining a flat or inert position while pups are attempting to nurse is considered lower quality maternal care (for a description, see Champagne *et al.* 2003; Peña & Champagne 2013). Nursing postures were not defined or categorised as such in the present study and have not previously been defined in mink, aside from postures that block access to teats entirely (Dawson *et al.* 2013). Investigation of the impact of nursing postures on kits’ ease of access to milk and correlation with licking and grooming behaviour may therefore be informative to assess in future studies of mink maternal care. However, it should also be noted that most existing research on the role of maternal nursing, licking, and grooming on long-term offspring fear behaviour and/or HPA-axis reactivity has featured species that are social. There is limited evidence that this particular mechanism applies in solitary carnivores like mink, and though there has been recent work demonstrating that early separation from the mother with or without sibling presence in cats (*Felis catus*) (a relatively solitary carnivorous species) affects later emotional reactivity of kits (Martínez-Byer *et al.* 2023), this study did not directly measure outcomes of early maternal separation or lessened maternal care on kit HPA-axis responsivity.

Relatedly, our prediction that pre- and post-stress faecal cortisol in EW kits would not significantly differ due to improvements in HPA-axis regulation was not supported. EW kits’ post-stress FCM concentrations were in fact significantly decreased compared to that of the pre-stress period, though this result also does not directly oppose our prediction since post-stress cortisol levels would be expected to increase if feedback sensitivity of the HPA axis was reduced. Moreover, there was no change in SH or EK kits’ faecal cortisol across these periods. These results are highly unexpected given that a previous study using these methods of cortisol sampling in mink did find increases in faecal cortisol following both a 15-min period of immobilisation in a carrying cage and a 2-h period of ‘handling’ in which mink were trapped, immobilised in a carrying cage, and repeatedly sampled for blood (Malmkvist *et al.* 2011).

However, in the present study, pelt grading was implemented as an alternative to prolonged immobilisation stress, which may be the source of this discrepancy. Pelt grading is a novel experience for kits and involves capture, restraint on a pelt grading table under a bright light, and manipulation of the pelt to assess hair nap, thus it was presumed to be sufficient to evoke a stress response (moreover, there is evidence from a previous study that fearful temperaments in mink increase following pelt grading; Bak & Malmkvist 2020). However, we cannot exclude the possibility that the experience was too brief to induce measurable changes in faecal cortisol as kits were typically returned to their pens after only one or two minutes of handling. A recent study was also unable to detect differences in minks’ faecal cortisol before and after 15-min immobilisation in a carrying cage and relocation to a new pen due to high individual variation in cortisol levels at each sampling point, and relatively high baseline means which the authors propose may have blurred cortisol responding due to a ceiling effect (Malmkvist *et al.* 2024). Thus, there may be high levels of variation in individual responses to handling or immobilisation stress of certain durations and intensities. Responses to these stressors may also vary between farm populations, as fear of humans has been known to do (Meagher *et al.* 2011), and fear traits are known to be highly heritable and subject to selection in mink (Hansen 1996; Malmkvist & Hansen 2001, 2002; Berg *et al.* 2002; Thirstrup *et al.* 2019). As discussed in the companion article to this study (Clark *et al.* 2025), relatively low levels of fear behaviours were exhibited by our subjects during pelt grading; approximately twenty percent of kits assessed for stress responsiveness demonstrated struggling, biting, or urination during handling, and fear vocalisations occurred in very low numbers on average (mean of 1.05 vocalisations across all housing conditions combined).

The pelt grading event may also have been perceived by kits as similar to past stressors (i.e. previous handlings for immunisations, weight recordings, or pen moves); rodent studies have demonstrated the potential for a high degree of adaptation of corticosterone responses after repeated exposure to acute stressors, particularly if these stressors occur in adolescence – perhaps as a result of the greater ability of juveniles to adapt their behaviour as a stress-coping strategy (Sadler & Bailey 2016; Papilloud *et al.* 2018). It could also be postulated that kits were demonstrating blunted HPA-axis responses to stress (hypo-responsiveness or non-responsiveness to stress can occur in cases of extreme chronic stress; Herman *et al.* 2016), though there was limited evidence of chronic stress markers in our subjects. For example, the spleens of males across all groups were relatively heavy, which is hypothesised to be indicative of greater ability to invest in lymphocyte production or storage and thus good health and low-stress conditions (Nunn 2002). However, we were presently unable to assess hormonal activity at other stages of the HPA-axis response (e.g. ACTH or CRH levels), nor other tissue-related measures of stress (e.g. adrenal weights), so speculation on this point is limited.

## Animal welfare implications

The materials provided to dams in enriched whelping conditions positively impacted behaviours associated with poor welfare (i.e. stereotypic behaviour) and may therefore be recommended as welfare-improving provisions on mink farms and in other contexts where nest-building carnivores are kept in captivity. Although we were unable to detect effects of this enrichment on basal cortisol levels of dams, further study with larger sample sizes or different sampling methods may be useful in determining whether these materials have additional physiological welfare benefits for dams. Further research on the influence of enriched whelping conditions on HPA-axis responsiveness of kits would also inform whether this short-term, relatively feasible housing intervention can improve kits’ long-term ability to cope with stressors.

## Conclusion

The nest-building materials and hanging rope provided in the EW housing condition positively impacted dams’ stereotypic behaviour and significantly improved nest shapes in the perinatal period. Maternal stress and maternal care delivered to kits did not appear to be impacted by this intervention, though EW dams may have been more efficient in their kit-directed maternal care behaviour. Since nursing and grooming of kits were not performed at higher levels in EW dams, it follows that EW kit stress responsiveness did not appear to be affected; though, the stress event used for this test may have been inadequate to observe an HPA-axis response due to insufficient restraint durations or habituation of kits’ cortisol responses to repeated handling. In terms of potential on-farm applications of these enrichment strategies, this study demonstrates that dams will utilise additional nest-building materials (particularly crumpled paper tissue) and will unwind hanging rope enrichments to weave into their nests. Benefits of these materials to other aspects of maternal welfare as well as maternal care and long-term kit welfare should be further investigated.
